# Non-coding RNA in cancer

**DOI:** 10.1042/EBC20200032

**Published:** 2021-10-27

**Authors:** Huiwen Yan, Pengcheng Bu

**Affiliations:** 1 Key Laboratory of RNA Biology, Key Laboratory of Protein and Peptide Pharmaceutical, Institute of Biophysics, Chinese Academy of Sciences, Beijing 100101, China; 2 College of Life Sciences, University of Chinese Academy of Sciences, Beijing 100049, China; 3 Center for Excellence in Biomacromolecules, Chinese Academy of Sciences, Beijing 100101, China

**Keywords:** cancer, circular RNA, long non-coding RNA, microRNA, non-coding RNA, piwi RNA

## Abstract

Majority of the human genome is transcribed to RNAs that do not encode proteins. These non-coding RNAs (ncRNAs) play crucial roles in regulating the initiation and progression of various cancers. Given the importance of the ncRNAs, the roles of ncRNAs in cancers have been reviewed elsewhere. Thus, in this review, we mainly focus on the recent studies of the function, regulatory mechanism and therapeutic potential of the ncRNAs including microRNA (miRNA), long ncRNA (lncRNA), circular RNA (circRNA) and PIWI interacting RNA (piRNA), in different type of cancers.

## Introduction

**Figure 1:**
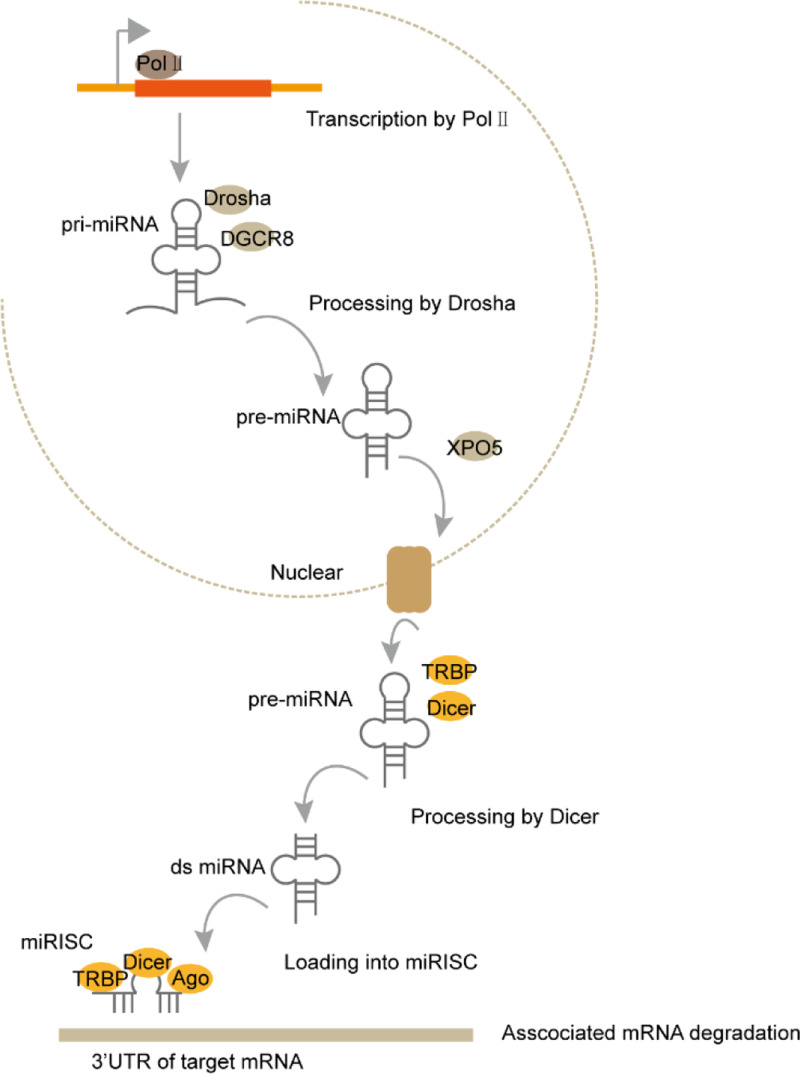
The biogenesis and effector machineries of miRNAs miRNAs are transcribed as pri-miRNAs by RNA polymerase II. Following processing by the Drosha complex, pre-miRNAs are exported to the cytoplasma by exportin 5 (XPO5). Mature miRNAs are produced by Dicer and TAR RNA-binding protein 2 (TARBP2)-mediated processing and loaded into the RISC. miRNAs function through degrading mRNA or repressing translation to regulate cancer.

**Figure 2:**
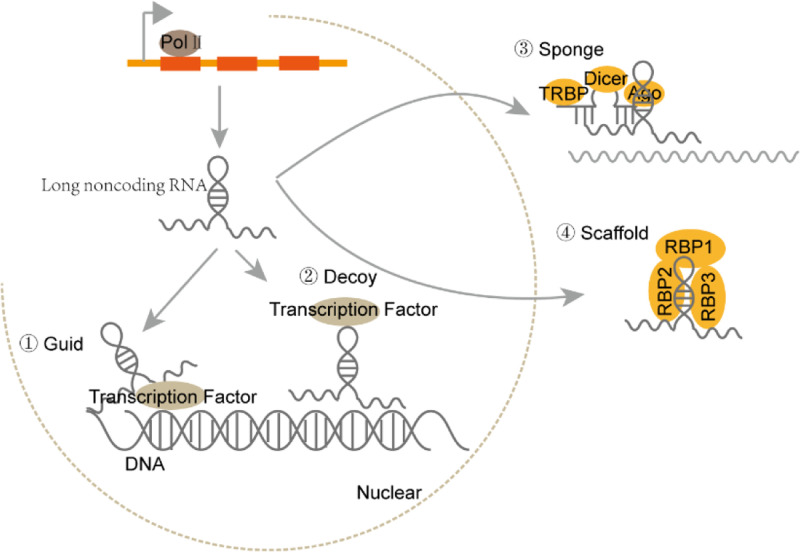
The biogenesis and effector machineries of lncRNAs LncRNAs are transcribed by RNA polymerase II. LncRNAs function as guide molecules to recruit factors for chromatin remodeling, as decoys to hinder transcriptional factors from the promoter of target gene, as sponges of associated miRNA to prevent degradation of target gene, or as scaffolds to facilitate interaction of associated proteins.

**Figure 3:**
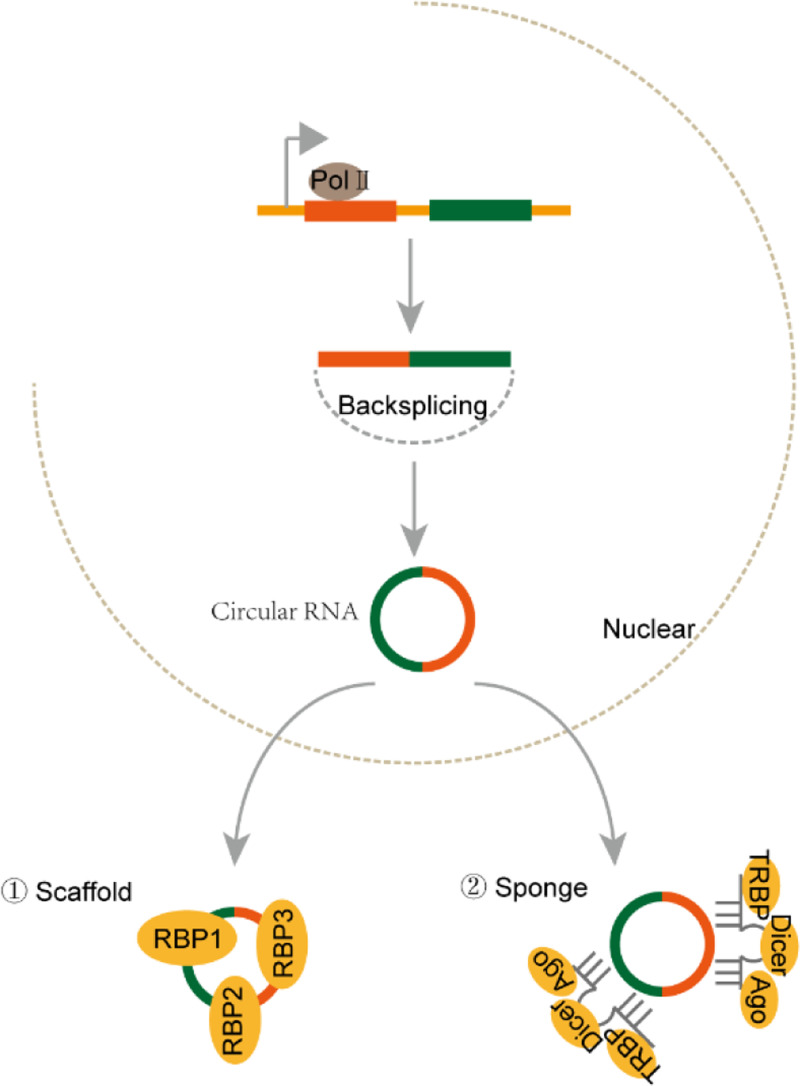
The biogenesis and effector machineries of circRNAs circRNAs are transcribed by RNA polymerase II and cyclized by backsplicing. circRNAs function as scaffolds to facilitate interaction of associated proteins, or as miRNA sponges to prevent degradation of target gene.

Approx. 75% of the human genome is transcribed into RNA, while only 3% is transcribed into protein-coding mRNAs [1]. According to the length, shape and location, non-coding RNAs (ncRNAs) have been divided into different classes. Among them, microRNA (miRNA), long ncRNA (lncRNA), circular RNA (circRNA) and PIWI interacting RNA (piRNA) are the four major ncRNA types with distinct functions in cancers. miRNAs are a kind of small RNA with approx. 22 nucleotides (nt) in length. miRNAs bind to the complementary sequence in targeted mRNA and cause RNA-induced silencing complex (RISC) to degrade targeted mRNA (Figure 1) [2]. piRNA was first identified in *Drosophila* with 24–30 nt in length. It mainly exists in germline cells and binds to PIWI family proteins to participate in epigenetic regulation of chromatin [3]. LncRNAs and circRNAs are more than 200 nt long, but lncRNAs are linear, while circRNAs are ringlike. Both lncRNAs and circRNAs can be transcribed from exon, intron, intergenic region or 5′/3′-untranslational regions and fold into complicated second structures, which facilitate their interactions with DNA, RNA and proteins (Figures 2 and 3) [4]. LncRNAs and circRNAs regulate gene expression through multiple mechanisms. They can play as miRNA decoy to prevent the targeted mRNA degradation. They can modulate transcription factors to bind to promoters and thus regulate targeted gene expression [5]. They can also work as scaffold to regulate protein–protein interactions and the related downstream signaling pathways. Recently, some studies showed that lncRNAs and circRNAs participated in epigenetic modulation of chromatin to regulate gene expression.

Abundant evidences have shown that ncRNAs play crucial roles in human malignancies. They can work as oncogenes or suppressors to regulate cancer initiation and progression. Many ncRNAs can be released from cancer cells into blood or urine and act as diagnostic markers or prognostic indicators. Here, we mainly focus on overviewing the recently emerging studies of the four major ncRNAs in cancer.

## miRNAs in cancers

Numerous studies have shown the important role of miRNAs in various cancers. Many miRNAs are highly expressed in cancer cells and promote cancer development. Some miRNAs even regulate the progression of multiple cancers. miR-126 is known to be highly expressed in breast [6] and colorectal cancers [7]. Recently, Silva et al. showed that miR-126 was also highly expressed in human B-ALL [8]. Forced expression of miR-126 in mouse hematopoietic stem progenitor cells resulted in B-cell leukemia. Further study revealed that overexpression of miR-126 down-regulated the expression of p53 and its associated genes [9], while suppression of miR-126 triggered apoptosis and inhibited B-ALL progression in xenograft mice. miR-155 has been identified as an oncogene in many kinds of cancers, including colon, breast, lung, gastric and liver cancer [10–14]. In agreement with its oncogenic roles, miR-155 has been regarded as a therapeutic target in different cancers. Recently, miR-155 was further shown to be up-regulated in plexiform neurofibromas [15]. Up-regulated miR-155 increased proliferation and sphere formation of plexiform neurofibromas initiating cells. Inversely, anti-miR-155 nucleic acid decreased tumor number in mouse spontaneous plexiform neurofibromas model. miR-215 is another oncogene and up-regulated in glioblastoma by hypoxia [16]. Hypoxia-elevated miR-215 targets epigenetic regulator KDM1B, to regulate the related downstream signaling and thus maintain glioblastoma initiating cell growth [17]. Some miRNAs, such as miR-105 can be secreted by cancer cells via exosome to modulate tumor microenvironment. miR-105 is highly expressed in metastatic breast cancer cells [18]. After secretion, miR-105-containing exosomes enter into endothelial monolayers and suppress the expression of the tight junction protein ZO-1, resulting in elevated vascular permeability and cancer metastasis [18]. Zhuo et al. further showed that circulating miR-105 could act as a clinical indicator of breast metastasis.

Some miRNAs have been regarded as tumor suppressors, such as let-7 and miR-34a. The let-7 miRNAs contain many family members. Most of them are down-regulated in different types of cancers, including hepatocellular carcinoma [19], non-small cell lung cancer [20], prostate cancer [21], breast cancer [22], colon cancer [23] and pancreatic cancer [24]. Let-7 miRNAs target and down-regulate many oncogenic genes including E2F1, ARID3B, K-RAS and c-Myc, resulting in suppression of tumor progression [25]. Furthermore, higher levels of let-7 indicate better prognosis in hepatocellular carcinoma and thyroid carcinoma [26]. Recently, Pablo et al. showed that let-7 also targeted Long Interspersed Element class 1 (LINE-1), the only autonomously active transposable elements highly expressed in lung cancer, to impair its translation and reduce its mobilization [27]. They proposed that Let-7 sustained somatic genome integrity by restricting LINE-1 retrotransposition. miR-34a is another tumor suppressor that plays an important role in suppressing cancer progression. We previously showed that miR-34a was critical for asymmetric division of colon cancer stem cells (CCSCs) [28]. Silencing miR-34a inhibits asymmetric cell division, promotes CCSC self-renewal and thus accelerates colon cancer progression. Kennerdell et al. also showed that miR-34a was decreased in most of the colon cancer cell lines and low levels of miR-34a predicted poor prognosis [29]. Tumor suppressor miR-29 is identified in microenvironment of chronic lymphocytic leukemia (CLL). In CLL, miR-29 targets Tumor-Necrosis Factor (TRAF4), a factor associated with CD40 activation and B-cell receptor signaling [30]. Down-regulated miR-29 elevates the expression of TRAF4 and activates CD40 signaling in CLL. Reversely, activated CD40 represses the expression of miR-29. miR-29-TRAF4-CD40 signaling axis plays as a negative feedback regulation loop in CLL. We have summarized the recent studies on miRNA functions in cancer in Table 1.

**Table 1:** List of miRNAs and their role in cancer development

Cancer type	Oncogene			Tumor suppressor		
**Breast**	let-7	sustains self-renewing	[73]	miR-30	promotes apoptosis	[76]
	miR-141	promotes metastasis	[74]	miR-140	inhibits proliferation	[77]
	mi-766	promotes proliferation, chemoresistance, migration and invasion	[75]	miR-143	inhibits proliferation	[78]
				miR-600	inhibits stemness	[79]
				miR-7	inhibits cell growth	[80]
**Lung**	miR-518b	promotes proliferation and metastasis	[81]	let-7	represses expression of k-Ras	[83]
	miR-629	promotes proliferation and metastasis	[82]	miR-200a	represses EMT	[84]
				miR-190b	suppresses cell growth	[85]
**Ovarian**	let-7	elevates multiple drug resistance	[86]	miR-134-3p	reduces multiple drug resistance	[87]
				miR-126	inhibits proliferation	[88]
**Prostate**	miR-141	promotes proliferation	[89]	miR-145	inhibits proliferation and invasion	[90]
				miR-34	reduces stemness	[91]
**Colorectal**	miR-1274a	promotes proliferation and metastasis	[92]	miR-137-3p	inhibits migration	[94]
	miR-592	promotes proliferation and clonogenicity	[93]	miR-22	represses invasion	[95]
				miR-3622a-3p	reduces stemness	[96]
**Brain**	miR-137	promotes proliferation	[97]	miR-128	inhibits proliferation and differentiation	[98]
				miR-136	promotes apoptosis	[99]
**Pancreatic**	miR-200b-3p	sustaining self-renewing	[100]	miR-142-5p	inhibits proliferation	[101]
**Liver**	miR-93-5p	suppresses senescence	[102]	miR-342-3p	inhibits proliferation	[103]
				miR-1225-5p	inhibits proliferation and invasion	[104]
				miR-589	suppresses stemness	[105]
**Stomach**				miR-635	inhibits proliferation and invasion	[106]
				miR-876-5p	inhibits proliferation and invasion	[107]
**Leukemia**	miR15/16	Sustains stemness	[108]	miR-99	suppresses stemness	[109]
				miR-185	impairs survival of drug-resistant cells	[110]
				miR-146a	alleviates myeloma proliferation	[111]

## lncRNAs in cancers

Like miRNAs, lncRNAs also play as oncogenes or suppressors to regulate tumorigenesis and progression. HOTTIP, derived from *HOXA* gene, has been shown to be highly expressed in many caners. Recently, Luo et al. demonstrated that HOTTIP played as an oncogene in acute myeloid leukemia (AML) [31]. They found that HOTTIP was aberrantly elevated in AML and worked as an epigenetic regulator to modulate hematopoietic gene-associated chromatin signature and transcription. LncTCF7 is another lncRNA transcribed from TCF gene locus. Wang et al. showed that lncTCF7 was highly expressed in liver cancer stem cells (CSCs) and was important for liver CSC self-renewal [32]. Mechanistically, LncTCF7 recruited SWI/SNF complex to TCF7 promoter and activated Wnt signaling for sustaining liver CSC self-renewal. Epigenetically induced lncRNA1 (EPIC1) is first identified as an oncogene in luminal B breast cancer [33]. Recently, EPIC1 has been found to be highly expressed in glioma [34], cholangiocarcinoma [35], pancreatic [36] and lung cancers [37]. Elevated EPIC1 promotes tumor growth by interacting with MYC to elevate its target genes, such as *CDKN1A*, *CCNA2* and *CDC20* [33]. Recently, Li et al. showed that linc0624, an antisense strand of CHD1L, worked as molecular decoy to segregate HDAC6–TRIM28–ZNF354C transcriptional corepressor complex away from the specific genomic loci, thus promoting the progression of hepatocellular carcinoma [38].

Some lncRNAs act as suppressors to suppress cancer development and progression. Pvt1b, a p53-dependent isoform of the lncRNA, suppresses lung cancer growth by down-regulating c-Myc expression [39]. DIRC3 is down-regulated in melanomas and its lower expression level is associated with shorter survival [40]. Further study reveals that DIRC3 inhibits proliferation of melanoma cells via elevating the expression of tumor suppressor IGFBP5. Recently, SATB2-AS1, an antisense transcript of tumor suppressor SATB2, has also been shown to be down-regulated in colorectal cancer. Knockdown of SATB-AS1 significantly increases cell proliferation, migration and invasion [41]. Mechanistically, SATB-AS1 works as a scaffold to recruit p300 to SATB2 promoter, up-regulating SATB2. Elevated SATB2 recruits HDAC1 to Snail promoter, suppressing Snail expression and epithelial-to-mesenchymal transition. MALAT1, a nuclear lncRNA, is also a tumor suppressor in breast cancer. Jong et al. showed that knockout of MALAT1 promoted breast cancer metastasis through disrupting the recruitment of transcription factor TEAD and co-activator YAP to the target gene promoters [42]. We have summarized the recent studies on lncRNA functions in cancer in Table 2.

**Table 2:** List of lncRNAs and their role in cancer development

Cancer type	Oncogene			Tumor suppressor		
**Breast**	00617	promotes metastasis	[112]			
	XIST	promotes proliferation and inhibit apoptosis	[113]	SCIRT	restrains transcriptional program of tumor-initiating cells	[121]
	H19	promotes stemness	[114]			
	ROR	elevates multiple drug resistance	[115]	PVT1	inhibits cell growth	[122]
	HOTAIR	promotes proliferation and metastasis	[116]			
	01271	promotes metastasis	[117]			
	DILA1	promotes proliferation and multiple drug resistance	[118]			
	ERINA	promotes cell-cycle progression	[119]			
	TROJAN	promotes proliferation and invasion	[120]			
**Ovarian**	HOTAIR	promotes stemness	[123]			
	LINP1	promotes proliferation and invasion	[124]			
**Brain**	HAS2-AS1	promotes invasion	[125]	ROR	inhibits proliferation	[129]
	H19	promotes angiogenesis	[126]			
	CRNDE	promotes proliferation and invasion	[127]			
	XIST	promotes proliferation and invasion	[128]			
**Liver**	HOTAIR	promotes proliferation and invasion	[130]	DILC	suppresses stemness	[136]
				PTENP1	suppresses proliferation and invasion	[137]
	β-Catm	sustains self-renewing	[131]			
	TRG-AS1	promotes proliferation and invasion	[132]	TSLNC8	suppresses proliferation and metastasis	[137]
	HUR1	promotes proliferation	[133]		inhibits cell growth, cell survival and transformation	[138]
	01138	promotes proliferation, invasion and metastasis	[134]	TCAM1P-004	inhibits cell growth, cell survival and transformation	[138]
	MALAT1	promotes proliferation and inhibit apoptosis	[135]	RP11-598D14.1		
**Colon**	URHC	promotes proliferation and invasion	[139]	PGM5-AS1	inhibits proliferation and invasion	[142]
	CCAT2	elevates chromosomal instability and promote proliferation and invasion	[140]	00959	suppresses migration and invasion	[143]
	PURPL	promotes cell growth	[141]			
**Lung**	TRINGS	protects cancer cells from necrosis	[143]	00261	active DNA damage response and block proliferation	[146]
	MIR22HG	promotes cell survival	[144]			
	GUARDIN	sustains genomic stability and prevent apoptosis and senescence	[145]			
**Leukemia**	CRNDE	promotes proliferation	[147]	PANDA	inhibits cell growth	[148]

## circRNAs in cancers

circRNAs are recently identified ncRNA type and act as either tumor suppressors or oncogenes. For instance, circCDYL is down-regulated in colon cancer, bladder cancer and triple-negative breast cancer and its underexpression is positively correlated with patient survival [43]. Further studies shows that overexpression of circCDYL promots apoptosis and inhibits proliferation of breast cancer cells [44]. Mechanically, circCDYL functions as a sponge to protect TP53INP1 from miR-190a-3p-mediated down-regulation [45]. The expression of circFOXO3 is lower in the breast cancers compared with that in adjacent benign tissues [46]. Interestingly, circFOXO3 works not only as an miRNA sponge to protect Foxo3 mRNA from attack, but also as a scaffold to bridge p21 and CDK2 to inhibit cell cycle progression [47].

In contrast with the tumor suppressive roles, some cirRNAs have been identified as oncogenes. circ-CCAC1, also known as cholangiocarcinoma-associated circular RNA1, is highly expressed in cholangiocarcinoma and cholangiocarcinoma-derived endothelial vessels [48]. In tumor cells, circCCAC1 recruits miR-514a-5p to up-regulate YY1 and its downstream gene *CAMLG*, which elevates the cell activity [48]. In endothelial vessels, circ-CCAC1 up-regulates SH3GL2 by sequestering EZH2, thus reducing intercellular junction protein levels and increasing cell leakiness [48]. circRNAHIPK3 derived from exon 2 of *HIPK3* gene is highly expressed in many types of cancer, including glioma [49], prostate cancer [50], breast cancer [51], colorectal cancer [52] and renal cancer [53]. Through screening of 424 miRNAs, 9 miRNAs showed great suppressive ability on the HIPK3 exon 2. Interestingly, all the nine miRNAs have been identified as tumor suppressors and suppressed by circHIPK3 [54]. These studies demonstrate that the expression of circRNAs is dynamically regulated in different cancers, and regulates cancer progression through distinct mechanisms. We have summarized the recent studies on circRNA functions in cancer in Table 3.

**Table 3:** List of circRNAs and their role in cancer development

Cancer type	Oncogene			Tumor suppressor		
**Breast**	UBE2D2	elevates multiple drug resistance	[149]	000554	represses EMT	[152]
				HIPK3	inhibits proliferation and invasion	[153]
	DCAF6	sustains stemness	[150]			
	DNMT1	activates autophage	[151]			
**Lung**	MYLK	promotes glycolysis and proliferation	[154]			
	CPA4	promotes stemness	[155]			
	LDLRAD3	promotes proliferation and survival	[156]			
**Colon**	UBAP2	promotes proliferation and metastasis	[157]			
**Brain**	POSTN	promotes proliferation and metastasis	[158]	SHPRH	suppresses proliferation	[159]
**Liver**	0000517	promotes glycolysis and clonogenicity	[160]			
	0067934	promotes proliferation and metastasis	[161]			
	ASAP1	promotes proliferation, colony formation migration and invasion	[162]			
	CDYL	sustains stemness	[163]			
	10720	promotes EMT	[164]			
**Gastric**	0000144	promotes proliferation and clonogenicity	[165]			
	NRIP1	promotes proliferation and glycolysis	[166]			
**Ovarian**	FGFR3	promotes proliferation and EMT	[167]	9119	suppresses proliferation	[169]
				ITCH	suppresses proliferation, invasion and glycolysis	[170]
	UBAP2	promotes proliferation and inhibits apoptosis	[168]			
				MTO1	suppresses proliferation and invasion	[171]

## piwiRNAs in cancers

Generally, piRNAs are expressed in the germline, but recent studies have demonstrated that piRNAs are also expressed in cancer cells, where piRNAs play crucial role in repression of transposable elements cleaving, deadenylation and decay. For instance, piRNA-823 has been identified to regulate proliferation and migration of a variety of cancer cells [55,56]. In multiple myeloma (MM), silencing piRNA-823 induces the expression of apoptosis-related genes by modulating *de novo* DNA methylation [57]. In colorectal cancer, inhibition of piR-823 suppresses cell proliferation and induces cell apoptosis by activating apoptosis-associated transcription factor HSF1 [58]. Cordeiro et al*.* examined several piRNA pathways in classical Hodgkin lymphoma and found that piR-651 was down-regulated in classic Hodgkin lymphoma patients compared with that in healthy controls. In addition, low levels of piR-651 are positively correlated with short overall survival of the classic Hodgkin lymphoma patients [59]. piRNA-54265 is highly expressed in cancer tissue and serum of the colorectal cancer patients. piRNA-54265 activates STAT3 signaling by facilitating PIWIL2/STAT3/SRC complex assemble [60]. Thus, piRNAs are also important for cancer progression.

## Targeting ncRNAs in cancer therapy

Recently, several ncRNAs have been used as novel therapeutic targets to treat cancers. Considering different roles of ncRNAs in specific cancer types, ncRNA mimics, antisense oligonucleotides (ASOs) or small molecule drugs have been applied for the treatment of cancers. miR-34a mimic packaged in a liposomal nanoparticle, called MRX34, has gone through a phase I clinical trial in patients with advanced solid tumor [61]. Moreover, miR-31-3p and miR-31-5p have been considered as colorectal cancer predictive biomarkers in phase III clinical trial [62,63]. Li et al. took a computational approach to design and identify small molecules on the base of the predicted miRNA hairpin precursor structures. They found that a benzimidazole analog selectively inhibited the processing of pri-miR-96 into oncogenic miR-96 and thus elevated miR-96 target gene expression and promoted cancer cell apoptosis [64]. Further optimization of benzimidazole turns out a dimeric benzimidazole and bisbenzimide compound, targaprimir-96, which shows a favorable pharmacokinetics profile and is effective at releasing tumor burden in a triple-negative breast cancer xenograft mouse model [65]. Another dimeric benzimidazole and bisbenzimide analog, targaprimir (TGP)-515, is identified to target pri-miR-515, resulting in up-regulation of human epidermal growth factor receptor 2 and enhancement of the therapeutic efficacy of the anti-human epidermal growth factor receptor 2 antibody in breast cancer cells [66]. Likewise, a bisbenzimide analog called targarpremir-210, also called TGP-210, is identified to bind to pre-miR-210, leading to the inhibition of processing of mature miR-210 and suppressing the outgrowth of xenograft tumors in mice [67]. The attachment of a nuclease recruitment module on to targarpremir-210 offers a conjugate, TGP-210-RL, which is able to recruit RNase L on to pre-miR-210 to induce the degradation of pre-miR-210. Compared with TGP-210, TGP-210-RL conjugate exhibits higher binding affinity to the pre-miR-210 while lower affinity to DNA [68]. Recently, an oligonucleotide inhibitor of miR-155, called cobomarsen, has been reported to decrease cell proliferation and induces cell apoptosis in Diffuse Large B-cell Lymphoma. Clinically, this compound efficiently inhibits tumor growth without obvious side effects on the patients, supporting its potential therapeutic application in Diffuse Large B-cell or other types of Lymphoma [69]. Further computational and experimental studies demonstrates that mitoxantrone is able to directly bind to pre-miR-21 and subsequently inhibits Dicer-mediated biogenesis of oncogenic miR-21 [70]. Several studies have demonstrated that ASOs can be used as inhibitors to block lncRNAs [71]. In mouse model, ASOs targeting MALAT1 blocks metastasis of lung cancer cells [72]. Together, targeting ncRNAs has been showing a promising approach for cancer therapy.

## Conclusion

ncRNAs contain various classes and participate in regulation of the progression of various types of cancers. Some ncRNAs highly exist in serum or urine of the cancer patient and are capable to work as diagnostic markers or prognostic indicators. Many clinical trials have also been conducted by targeting ncRNAs and exhibited promising therapeutic effects. With deep investigation of the mechanisms, we have been broadening our understanding of ncRNA functions. For instance, miRNAs are originally considered to suppress target gene expression by binding to the 3′-UTR regions. Recently, we have realized that miRNAs could also bind to other regions of the genes and even up-regulate target gene expression. Now we also know that some lncRNAs actually can encode small peptides to regulate biological processes. However, there are still many unknown ncRNAs, particularly the new ncRNA classes with precise roles need to be investigated. Even for the well-known ncRNAs, their function and regulatory mechanisms could be changed with spatial-temporal alteration, such as expression pattern, structure and interacting proteins. Therefore, efforts still need to make to understand the precise function and mechanisms of the ncRNAs.

Targeting ncRNA therapies have been conducted in many clinical trials. Emerging technologies and new approaches will contribute to even better outcomes. For instance, targeting ncRNA approaches could be co-operated with immune therapy or other therapeutic treatments. Human organoids can be used for investigating functions or preclinical effects of ncRNAs in patients. Targeting ncRNAs by CRISPR-mediated gene editing may also be worth trying for certain diseases. Many ncRNAs both functions in physiology and pathology. Therefore, deep investigation of the function and mechanism will help to identify the ncRNAs specifically regulating cancers and reduce the adverse side effects. Overall, ncRNAs are heavily involved in regulating various cancers and targeting ncRNAs have exhibit promising therapeutic effect, while we still need to keep making efforts to reveal the mystery of ncRNA functions.

## Summary

- ncRNAs work as oncogenes or tumor suppressors to regulate carcinogenesis and progression.
- ncRNAs regulate cancer progression through distinct mechanisms and represent potential drug targets or therapeutic entities.
- Clinical trials have been conducted to treat cancers by targeting ncRNAs and exhibited promising therapeutic effect.

## Abbreviations

AML: acute myeloid leukemia
ASO: antisense oligonucleotide
CCSC: colon cancer stem cell
circRNA: circular RNA
CLL: chronic lymphocytic leukemia
CSC: cancer stem cell
EPIC1: epigenetically induced lncRNA1
LINE-1: long interspersed element class 1
lncRNA: long non-coding RNA
miRNA: microRNA
ncRNA: non-coding RNA
nt: nucleotide
piRNA: PIWI interacting RNA
TRAF4: tumor-necrosis factor 4
B-ALL: B cell acute lymphocytic leukemia
